# The Role of SARS-CoV-2 Nucleocapsid Protein in Host Inflammation

**DOI:** 10.3390/v17081046

**Published:** 2025-07-27

**Authors:** Yujia Cao, Yaju Wang, Dejian Huang, Yee-Joo Tan

**Affiliations:** 1 Department of Food Science and Technology, National University of Singapore, Singapore 117542, Singapore; yujia.cao@u.nus.edu (Y.C.); dejian@nus.edu.sg (D.H.); 2 Infectious Diseases Translational Research Programme, Department of Microbiology and Immunology, Yong Loo Lin School of Medicine, National University of Singapore, Singapore 117545, Singapore; 3 Biomedical and Health Technology Platform, National University of Singapore (Suzhou) Research Institute, Suzhou 215123, China

**Keywords:** SARS-CoV-2, nucleocapsid protein, biological function, host inflammation, long COVID

## Abstract

Severe acute respiratory syndrome coronavirus 2 (SARS-CoV-2), the causative agent of coronavirus disease 2019 (COVID-19), has posed substantial health threats and triggered widespread global economic disruption. The nucleocapsid (N) protein of SARS-CoV-2 is not only a key structural protein but also instrumental in mediating the host immune response, contributing significantly to inflammation and viral pathogenesis. Due to its immunogenic properties, SARS-CoV-2 N protein also interacts with host factors associated with various pre-existing inflammatory conditions and may possibly contribute to the long-term symptoms suffered by some COVID-19 patients after recovery—known as long COVID. This review provides a comprehensive overview of recent advances in elucidating the biological functions of the N protein. In particular, it highlights the mechanisms by which the N protein contributes to host inflammatory responses and elaborates on its association with long COVID and pre-existing inflammatory disorders.

## 1. Introduction

COVID-19 is caused by SARS-CoV-2, which is a positive-sense, single-stranded RNA virus. Millions of COVID-19 cases have been recorded globally, posing significant challenges for public health and inflicting considerable economic damage [1]. Similar to SARS-CoV and Middle East respiratory syndrome coronavirus (MERS-CoV), which are responsible for two drastic outbreaks, SARS-CoV-2 is a member of the *Coronaviridae* family [2]. Based on their genetic and serological features, coronaviruses (CoVs) are divided into four genera, namely α-, β-, γ-, and δ-coronaviruses [3]. Among these, α- and β-CoVs mainly infect mammals and pose serious threats to humans. SARS-CoV, MERS-CoV, SARS-CoV-2, human coronavirus (HCoV)-OC43, and HCoV-HKU1 are classified as β-CoVs; HCoV-229E and HCoV-NL63, on the other hand, are members of the alpha-CoVs [3].

The SARS-CoV-2 virion is primarily composed of the spike (S) protein, envelope (E) protein, membrane (M) protein, N protein, and viral RNA. Its genome contains 14 functional open reading frames (ORFs), with ORF1a and ORF1b occupying the majority of the genome and encoding 16 nonstructural proteins (NSPs) that are essential for viral replication. Additionally, nine accessory proteins contribute to host immune evasion and promote viral replication [4]. Importantly, the four structural proteins play significant functions in viral entry, assembly, and replication. Among them, the N protein plays key roles in viral mRNA transcription and replication, in addition to being involved in modulating immune responses and viral pathogenesis [5]. Therefore, it has been developed as a potential target for therapeutic development and diagnostic applications in COVID-19.

This review highlights recent findings on the modulatory functions of SARS-CoV-2 N protein across multiple stages of the viral life cycle, as well as its critical role in regulating host immune responses. In addition, the possible contribution of the N protein to long COVID and the interplay between the N protein and other pre-existing inflammatory diseases are also summarized.

## 2. N Protein

### 2.1. N Protein Structure

The SARS-CoV-2 N protein is a multidomain RNA-binding protein comprising 419 amino acids [6]. Two conserved and functional structural domains, the N-terminal domain (NTD) and C-terminal domain (CTD), are linked by intrinsically disordered regions (IDRs), including the central linking region (LKR), which contains a Ser/Arg (SR)-rich region that serves as a putative phosphorylation site. In addition, the N protein is flanked by two other LKRs, referred to as the N-arm and C-tail [6].

### 2.2. N Protein and Viral Life Cycle

SARS-CoV-2 entry into host cells is orchestrated by the S protein through endocytosis or direct fusion with the cell membrane [7]. After entry, the N protein is liberated into the cytoplasmic compartment, where viral genomic RNA dissociates from the N protein and undergoes replication and translation [8]. Newly synthesized viral genomes bound with N protein are assembled into enveloped particles with other structural proteins within the lumen of the endoplasmic reticulum–Golgi intermediate compartment (ERGIC) [8]. The progeny virions are then transported from the ERGIC to the Golgi apparatus and ultimately to the membrane, where they are released through exocytosis or lysosomal trafficking [8].

The SARS-CoV-2 N protein is essential for forming a helical ribonucleoprotein (RNP) complex with the RNA genome, mediated by regions with high positive charge on the surfaces of both NTD and CTD [9,10]. The RNP is crucial for viral replication and transcription, as it facilitates virion assembly and shields the virus from the dynamic immune response [9]. The interaction between the N protein and viral RNA, driven by liquid–liquid phase separation (LLPS), results in the formation of membraneless biomolecular condensates, which play key roles in multiple steps of the viral life cycle [11].

During LLPS, 5′-proximal genomic RNA transcripts of SARS-CoV-2 undergo conformational changes, mediated by the stem-loop (SL) SL1 and SL5a/b/c. These SL conformers are preferentially bound by the N protein [12]. Through LLPS, the N protein promotes the assembly of the RNA polymerase (RdRp) complex, including NSP7, NSP8, NSP12, and polyU RNA, ensuring high initiation and elongation rates during viral transcription [13]. A high-frequency trinucleotide polymorphism (GGG-to-AAC) variation leads to amino acid substitutions such as N^R203K/G204R^, significantly enhancing LLPS formation [14]. This mutation also promotes N protein phosphorylation and confers resistance to glycogen synthase kinase-3 (GSK-3) inhibition, thereby facilitating viral replication [15]. The N protein is recruited to the RTCs during genome replication by binding to the Ubl1 domain of NSP3, enhancing protein–RNA interactions either through enzymatic activities or by stabilizing the complex structure [16]. In early infection, phosphorylation of the SR-rich region of N protein by cytoplasmic kinase regulates the function of N protein. This modification facilitates RNA structural rearrangements required for the transcription of long subgenomic RNAs in the RTC by interacting with DEAD-box helicase 1 (DDX1) [17,18]. Additionally, it is discovered that the N-CTD plays a key part in interactions with the M protein during the budding process, as well as in forming the RNA-binding groove required for viral assembly [19]. The N protein also binds strongly to anionic lipids, such as phosphoinositide and phosphatidylserine on the membranes, via its CTD, which supports its localization to assembly sites [20]. The E3 ubiquitin ligase tripartite motif protein 6 (TRIM6) binds to N-CTD through its RING and B-box-CCD domains and modulates the K29-typed polyubiquitination of N-NTD (K102) and N-CTD (K347 and K361) [21]. The ubiquitination enhances the ability of N protein to bind to viral genomic RNA, thereby promoting viral propagation [21]. The N-C tail of N protein contributes to the encapsidation of genomic RNA, which may be regulated by interactions with the M protein [18]. Since viruses cannot produce ATP independently, they rely entirely on the host cell’s ATP as an energy source for key stages of their life cycle [22]. Intriguingly, the N-CTD has recently been identified as a novel ATP-binding site, suggesting its role in modulating phase separation [22].

## 3. N Protein and Inflammation

### 3.1. N Protein and Innate Immunity

#### 3.1.1. N Protein and Intracellular Inflammatory Signaling

**Figure 1:**
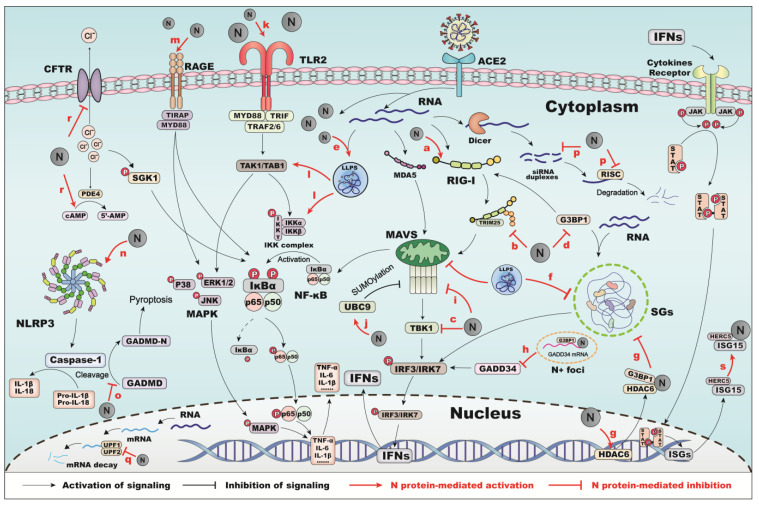
Proposed innate immune processes involving the N protein in host cells. Retinoic acid-inducible gene I (RIG-I) and melanoma differentiation-associated gene 5 (MDA5) are activated upon sensing dsRNA in the cytoplasm of infected cells. RIG-I and MDA5 initiate the mitochondrial antiviral signaling (MAVS) protein, thereby triggering a cascade of immune responses such as nuclear factor kappa B (NF-κB), interferon regulatory factor (IRF) 3, IRF7, and type I interferon (IFN-1) pathways. (**a**) SARS-CoV-2 N protein interacts with the RIG-I protein through the enzymatic active DExD/H domain, repressing the inflammatory response. (**b**) SARS-CoV-2 N protein interplayed with the TRIM25 and blocked RIG-I-mediated IFN production. (**c**) It also inhibited IFN signaling by targeting tank-binding kinase 1 (TBK1) and prevented the nuclear translocation of IRF3. Stress granules (SGs) are formed in response to viral infection as a host antiviral strategy. (**d**) SARS-CoV-2 N protein suppresses antiviral SG formation by interacting with GTPase-activating protein-binding protein 1 (G3BP1). (**e**) SARS-CoV-2 N protein undergoes LLPS with RNA during viral replication. (**f**) The formation of LLPS also inhibits SG assembly. (**g**) SARS-CoV-2 N protein also induces the expression of histone deacetylases 6 (HDAC6) to facilitate the interaction with G3BP1, thereby inhibiting the formation of SGs. (**h**) By sequestering growth arrest and DNA damage-inducible 34 (GADD34) mRNA into the N^+^foci and inhibiting its interaction with G3BP1, SARS-CoV-2 N protein further suppresses the transcription of IFN genes. (**i**) The SARS-CoV-2 N protein also suppressed the innate antiviral immune response by inhibiting Lys63-linked polyubiquitination and aggregation of MAVS. (**j**) SARS-CoV-2 N protein modulates MAVS SUMOylation by enhancing the interaction between UBC9 and MAVS. (**k**) SARS-CoV-2 N protein furthers the process of NF-κB signaling response and mitogen-activated protein kinase (MAPK) signaling pathway through Toll-like receptor 2 (TLR2). (**l**) During the LLPS process, N protein recruits key kinases of NF-κB signaling, such as TAK1 and IKK complex, increasing the possibility of the NF-κB activation. (**m**) The SARS-CoV-2 N protein binds to the receptor for advanced glycation end products (RAGE) via its NTD and CTD, activating the extracellular signal-regulated kinases 1 and 2 (ERK1/2)–NF-κB pathway. Nucleotide oligomerization domain-like receptor family pyrin domain containing-3 (NLRP3) functions in both inflammatory and antiviral responses. (**n**) SARS-CoV-2 infection promotes the activation of the NLRP3 inflammasome and caspase-1, (**o**) while GSDMD cleavage and pyroptosis are suppressed. RNA interference (RNAi) is a post-transcriptional gene silencing process. (**p**) SARS-CoV-2 N protein suppressed the RNAi in both initiation and effector steps by preventing siRNA biogenesis, RISC assembly, and target RNA cleavage, thereby evading the host inflammatory response. (**q**) Nonsense-mediated mRNA decay (NMD) is another antiviral mechanism involved in promoting mRNA regulation and degradation to prevent viral mRNAs translation. The SARS-CoV-2 N protein counteracts this mechanism by directly interacting with RNA helicase UP-frameshift-1 (UPF1) and UPF2. (**r**)The cystic fibrosis transmembrane conductance regulator (CFTR), a cAMP-dependent Cl^−^ channel, regulates the host immune defense against pathogen infection. SARS-CoV-2 N protein raises the intracellular Cl^−^ concentration by downregulating CFTR expression and depleting intracellular cAMP by increasing the phosphodiesterase 4 (PDE4), and the high intracellular Cl^−^ concentration induces the phosphorylation of serum glucocorticoid regulated kinase 1 (SGK1) and activates robust inflammatory responses. (**s**) SARS-CoV-2 N protein is ISGylated by HERC5 ISGylation.

The innate and adaptive immune systems are two major types of immune responses in the integrated human immune system. Innate immunity is activated when pattern recognition receptors (PRRs) detect viral pathogen-associated molecular patterns (PAMPs). Among the PRRs, the retinoic acid-inducible gene I (RIG-I)-like receptors (RLRs), including RIG-I and melanoma differentiation-associated gene 5 (MDA5), belonging to RLRs necessarily work in sensing cytoplasmic dsRNA during infection [23,24]. Upon activation, these receptors undergo conformational rearrangement and oligomerization, which initiates mitochondrial antiviral signaling (MAVS), thereby triggering a cascade of immune responses, such as the NF-κB, IRF3 and IRF7 pathways. These cascades ultimately lead to the production of type I interferon (IFN-I) and pro-inflammatory cytokines like IL-6 and IFN-α/β [25,26]. The N protein dampens host defenses and facilitates immune evasion by suppressing and modulating multiple components of the innate immune response (Figure 1).

RIG-I is a key cytosolic pattern recognition receptor that detects viral RNA and initiates the activation of IFN-I and other antiviral genes, thereby orchestrating the host innate immune response. TRIM25-mediated ubiquitination of RIG-1 is inhibited by the association between SARS-CoV N-CTD and the SPRY domain of TRIM25 [27]. Likewise, N protein associates with TRIM25, resulting in the inhibition of TRIM25-dependent IFN production [28]. N protein also inhibits polyinosinic: polycytidylic acid [poly(I:C)]-facilitated IFN signaling by targeting tank-binding kinase 1 (TBK1), thereby preventing IRF3 undergoing nuclear translocation [28]. Additionally, it interacts with the RIG-I protein via the enzymatically active DExD/H domain, repressing the IFN-β response [29]. A study demonstrated that mutations at R203 and D377 enhance the N protein-mediated impairment of RIG-I signaling, including reduced IRF3 phosphorylation and IFN-β release [30].

Stress granules (SGs) are cytoplasmic, electron-dense, membraneless structures formed in response to viral infection as part of the host’s antiviral defense mechanism [31]. The N protein has been proven to suppress antiviral SG formation by interacting with GTPase-activating protein-binding protein 1 (G3BP1), which has a critical function in RIG-I recognition of pathogenic RNA [32,33,34]. During viral replication, the N protein undergoes LLPS, which suppresses SG assembly and inhibits IFN-I production [34]. Moreover, the interaction between G3BP1 and the N protein is enhanced by N protein self-deacetylation, mediated through the induction of histone deacetylase 6 (HDAC6), a component of cytoplasmic SGs [35]. The methionine codon located at position 210 of the SARS-CoV-2 N protein (N^*M210^) is crucial for dsRNA binding and in inhibiting multiple aspects of the cellular antiviral response, such as SGs formation [36]. Furthermore, by sequestering growth arrest and DNA damage-inducible 34 (GADD34) mRNA into the N^+^foci and hindering its association with G3BP1, the N protein impedes IFN gene transcription and compromises the host innate immune response [37]. The SARS-CoV-2 N protein is known to interact with several proteins involved in immune responses, including activating signal co-integrator 1 complex subunit 3 (ASCC3), inosine monophosphate dehydrogenase 2 (IMPDH2), and adaptor-related protein complex 3 subunit beta 1 (AP3B1) [35].

Post-translational modification plays a pivotal role in regulating the effects of the SARS-CoV-2 N protein on the innate antiviral immune response. Previous research has shown that SARS-CoV-2 N protein represses the antiviral immune response by inhibiting Lys63-linked polyubiquitination and aggregation of MAVS. Furthermore, acetylation and deacetylation of Lys375 in the N-CTD region, mediated by host acetyltransferases and deacetylases, have been shown to inhibit MAVS signaling [38]. The N-CTD also serves a vital function in mediating liquid phase interaction with MAVS and inhibiting the MAV-mediated IFN response [39,40]. Poly (dA:dT), a RIG-I ligand, can induce the conjugation of the small ubiquitin-like modifiers (SUMO) to MAVS, thereby enhancing MAVS clustering and promoting the release of IFN-β. Suppression of the SUMOylation via ubiquitin-conjugating enzyme 9 (UBC9) siRNA significantly reduces the generation of poly(dA:dT)-induced IFN-β [41]. The SARS-CoV-2 N protein modulates MAVS SUMOylation by enhancing the interaction between UBC9 and MAVS, thereby interrupting IFN-β synthesis through suppression of IκB kinase-α (IKKα), TBK1, and IRF3 phosphorylation [42]. In addition, suppression of the innate antiviral immune response by the N protein is attributable to the SUMO conjugation on N protein lysine 65 residue. SUMOylation of N protein, facilitated by SUMO E3 ligase TRIM28, promotes its robust homo-oligomerization, RNA association, and LLPS activity [43]. Another E3 ligase, TRIM 21, mediates the polyubiquitination and degradation of N protein, thereby contributing to the regulation of host innate immunity [44]. Collectively, these findings demonstrate that post-translational SUMOylation of the N protein is critical for SARS-CoV-2 virulence and immune evasion. Surface proteins, including S, E, and M proteins, are easily glycosylated, while the N protein is less glycosylated but prone to phosphorylation [45]. Only two potential glycosylation sites on the N protein—N48 and N270—have been experimentally confirmed [46]. Glycosylation at these sites may mask epitopes, potentially interfering with host inflammatory responses; however, further evidence is still needed to substantiate these effects.

Furthermore, the underlying molecular mechanisms by which the SARS-CoV-2 N protein regulates the innate antiviral immune response have been increasingly elucidated, with NF-κB signaling emerging as one of the most prominent pathways activated in infected cells. Silencing NF-κB transcription factor p65 or p50 disrupts NF-κB signaling and reduces viral replication, an effect that is reversed upon their re-expression [47]. Intriguingly, N protein facilitates NF-κB response and cytokine production. Studies have found that N protein strongly activates NF-κB signaling pathways through toll-like receptor 2 (TLR2) and the mitogen-activated protein kinase (MAPK) pathway in human endothelial cells [48]. In HEK293T cells, N protein overexpression leads to cytoplasmic changes and increased nuclear accumulation of NF-κB p65 [49]. In murine macrophages, N protein not only regulates the secretion of pro-inflammatory cytokines, such as TNF-α, IL-6, and IL-10, but also alters the expression of immune-related genes in Janus kinase-signal transducer and activator of transcription (JAK-STAT), TNF, NF-κB, and MAPK signaling pathways [50]. A similar pattern of elevated cytokine expression is observed in N protein-stimulated lung epithelial A549 cells and the serum of COVID-19 patients [51]. The development of a cytokine storm, characterized by excessive production of cytokines such as IL-6 and poor type I IFN induction, is a hallmark of severe COVID-19 [52]. In murine models, N protein stimulation leads to acute lung injury marked by strong NF-κB activation and an exaggerated pro-inflammatory cytokine response [53]. Mechanistically, one reason for the interplay between N protein and NF-κB signaling may lie in the N protein’s ability to recruit key NF-κB pathway kinases, including TAK1 and IKK complex, during LLPS with RNA, thereby enhancing NF-κB activation [54]. In addition, the N protein binds to the receptor for advanced glycation end products (RAGE) via its NTD and CTD, activating the extracellular signal-regulated kinases 1 and 2 (ERK1/2)–NF-κB pathway through RAGE [55]. Interestingly, some genes show opposing expression patterns when NF-κB p65 is knocked down versus when the N protein is overexpressed, suggesting that N protein may trigger a distinct transcriptional program to modulate or fine-tune the NF-κB response [49].

Inflammasomes are multiprotein machinery that engage in innate antiviral immunity. They typically consist of adaptor apoptosis-associated speck-like protein-containing CARD (ASC), pro-caspase-1, and inflammasome nucleators such as nucleotide-binding oligomerization domain protein (NOD)-like receptors (NLRs), AIM2, and pyrin. Among the various identified inflammasomes, the NLR family pyrin domain containing-3 (NLRP3) inflammasome is the best understood because of its pivotal role in orchestrating inflammatory and antiviral mechanisms. Upon assembly, the NLRP3 inflammasome promotes pro-caspase-1 cleavage, which subsequently cleaves gasdermin D (GSDMD), inducing GSDMD-dependent pyroptosis. It also assists in the production of proinflammatory IL-18 and IL-1β [56]. The N protein has been shown to directly associate with NLRP3, enhancing its interaction with ASC and promoting NLRP3 inflammasome assembly. Remarkedly, lung damage and proinflammatory cytokine responses induced by the N protein can be mitigated by NLRP3 or caspase-1 inhibitors [57]. Another study demonstrated that N protein also directly interacts with the linker region of GSDMD, preventing its cleavage by caspase-1 and subsequently suppressing pyroptosis in SARS-CoV-2-infected human monocytes [58].

RNA interference (RNAi) plays critical antiviral defense roles in a wide range of organisms, including mammalian cells. It is a post-transcriptional gene silencing process initiated by double-stranded RNA (dsRNA), which is cleaved into small interfering RNA (siRNA) duplexes by the Dicer, an enzyme from the RNase III family of ATP-dependent ribonucleases [59,60]. At the effector phrase, these siRNA duplexes are incorporated into the RNA-induced silencing complex (RISC), guiding gene expression silencing [61]. The N protein suppresses the RNAi at both initiation and effector stages by obstructing siRNA biogenesis, RISC assembly, and target RNA cleavage. This enables the virus to evade host inflammatory responses and promotes viral replication in host cells [61]. In SARS-CoV, residue Lys 258 and Lys 262 with a positive charge in N-CTD were identified as critical residues for viral suppressors of RNAi (VSRs) [62]. A similar VSR region is presumed to exist in the N protein, likely within the CTD, though the exact residues remain unidentified. Another important antiviral mechanism is nonsense-mediated mRNA decay (NMD), which regulates and degrades aberrant mRNA to prevent the translation of viral transcripts. The N protein counteracts NMD by directly interacting with RNA helicase UP-frameshift-1 (UPF1) and UPF2, which are essential components in NMD. This interaction restrains the unwinding activity of UPF1 and disrupts the UPF1–UPF2 interaction, thereby inhibiting NMD and facilitating viral gene expression [63].

The cystic fibrosis transmembrane conductance regulator (CFTR) is a cAMP-dependent Cl^−^ channel that regulates the host immune defense against pathogen infection, such as SARS-CoV-2 [64]. The N protein downregulates CFTR expression by directly interacting with Smad3 via microRNA-145, leading to elevated intracellular Cl^−^ levels. In addition, the N protein further increases the intracellular Cl^−^ concentration by depleting intracellular cAMP by increasing the expression of phosphodiesterase 4 (PDE4). Consequently, the high concentration of intracellular Cl^−^ triggers serum glucocorticoid-regulated kinase 1 (SGK1) phosphorylation, subsequently activating a vigorous inflammatory response [65].

IFN-stimulated gene 15 (ISG15) is a ubiquitin-like protein that becomes covalently conjugated to host and viral proteins through intracellular enzymes such as HECT and RLD domain containing E3 ubiquitin protein ligase 5 (HERC5) [66]. The ISGylation is highly associated with the innate immune response against several viruses [67]. The N protein, particularly in its phosphorylated form, is subject to ISGylation by HERC5. This modification disrupts the N protein’s ability to form functional oligomers, ultimately inhibiting viral RNA synthesis [68]. Residues K261, K266, K355, K374, K387, and K388 within the N-CTD and spacer B/N3 domain are responsible for the ISGylation of N protein [67,68,69]. Interestingly, this antiviral ISGylation mechanism is counteracted by the SARS-CoV-2 papain-like protease (PLpro) through its deISGylating activity, thereby contributing to host antiviral immunity evasion [67,68].

The lectin pathway is a vital component of innate immunity, responsible for initiating the complement system, which is an immune surveillance mechanism that enables rapid detection and response to infections [70]. One study demonstrated that N protein aggravates inflammation by promoting overactivation of the lectin pathway through mannan-binding lectin serine protease 2 (MASP-2) overactivation [71]. However, other studies have shown that the N protein binds to neither MASP-1 nor MASP-2, nor does it activate the lectin pathway in normal human serum; instead, it undergoes proteolytic degradation when incubated with MASP-2 [72,73].

Although the N protein predominantly localizes in the cytoplasm, it is also detectable in the nucleus of infected cells, where it regulates gene expression and interferes with immune responses. Its nuclear translocation occurs via the nuclear pore complex and is mediated by interactions with Ras-related nuclear protein (RAN) and import receptors karyopherin alpha (KPNA) and karyopherin beta 1 (KPNB1) [66].

Importantly, the regulation of the innate immune response by the N protein is dose-dependent: a low concentration of the N protein inhibits IFN-I signaling and the secretion of inflammatory markers, while a high concentration of the N protein enhances the IFN-I pathway and the release of pro-inflammatory markers [74]. As SARS-CoV-2 evolves, subvariants with enhanced immune evasion capabilities tend to become dominant due to their ability to reduce innate immune activation. In response, the host undergoes convergent evolution to enhance innate immune antagonist expression, thereby counteracting viral immune escape [75].

#### 3.1.2. N Protein and Systemic Inflammation

**Figure 2:**
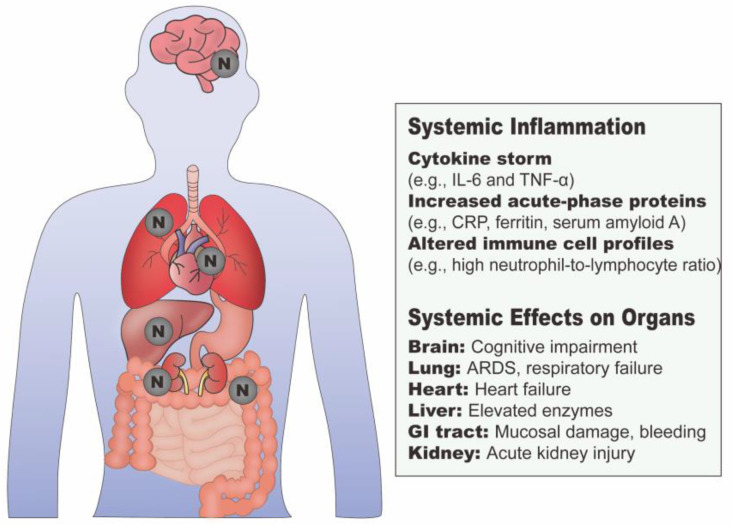
N protein-mediated systemic inflammation.

SARS-CoV-2 infection also triggers body-wide systemic inflammation, which can damage tissues and organs if not controlled. The influence of the N protein on systemic inflammation is illustrated in Figure 2. A drastic cytokine storm, characterized by considerably increased IL-6, IL-8, IL-10, TNF-α, and IFN levels in COVID-19 patients, leads to systemic inflammatory immune responses [76]. N protein induces the production of tumor necrosis factor receptor 2 (TNFR2), CCL4, IL-1β, IL-6, IL-6R, IL-10, and IL-12 in human monocytes [77]. Notably, N protein treatment results in the escalation of IL-6 mRNA expression, which might be attributable to the increased levels of NF-κB pathway markers like NF-κB subunit 1 (NFKB1) and NF-κB inhibitor alpha (NFKBIA) [77]. The LLPS of N protein also activates NF-κB and further facilitates robust secretion of IL-6, IL-1β, TNF-α, and CXCL10 [54]. While inflammasome activation does not directly drive the cytokine storm, it triggers the release of IL-1β, which, in turn, induces the production of IL-6 and IL-8 [78]. Mechanistically, pathogenic T helper type 1 (Th1) cells and CD14^+^CD16^+^ monocytes trigger proinflammatory signaling, leading to macrophage and neutrophil infiltration and a subsequent cytokine storm in the lungs [79]. IL-6 is a key cytokine in the COVID-19 cytokine storm and serves as an independent predictor of lung injury severity [80]. Its signaling has been linked to coagulopathy and hepatic endotheliopathy in COVID-19 pneumonia, contributing to the progression of liver injury [81].

Plasma levels of SARS-CoV-2 N antigen are associated with RAGE, IL-10, and IFN-gamma-inducible protein 10 kD (IP-10) levels, suggesting a role in modulating the progression to severe disease in hospitalized COVID-19 patients [82]. In a study involving 326 patients, 95% had plasma SARS-CoV-2 N antigen levels of at least 3 pg/mL, and 50% had levels equal to or exceeding 1000 pg/mL [83]. In another study, N protein concentrations in blood samples had a mean of 1734 pg/mL among 131 PCR-positive inpatients, compared to a mean of 157 pg/mL among 43 PCR-positive outpatients, indicating significantly higher N protein levels in hospitalized patients [84]. It has been demonstrated that the median plasma N antigen level is 7673 pg/mL in severe COVID-19 patients, 351.6 pg/mL in moderately affected patients, and 4.6 pg/mL in asymptomatic individuals [85]. The primary clinical outcome indicates that each 500 pg/mL increase in plasma N antigen level is associated with elevated inflammatory markers [82]. The exact mechanism by which the N protein exits the cell remains unclear; however, one plausible explanation is programmed inflammatory cell death, which may facilitate the release of intracellular contents, including unassembled viral proteins, through cell membrane rupture [86]. Extracellular vesicles, produced by most living cells, have the ability to transport viral proteins within a spherical lipid bilayer and are commonly found in blood, plasma, and serum, suggesting a possible mechanism for the dissemination of the N protein to the extracellular environment [87].

Clinically, COVID-19 patients admitted to the intensive care unit (ICU) exhibit elevated serum SARS-CoV-2 N antigen levels, which are associated with tissue damage biomarkers and heightened lung inflammatory responses [88,89]. C-reactive protein (CRP), produced in response to inflammation, activates the complement system and enhances phagocytosis, promoting the clearance of pathogens [90]. Elevated serum CRP levels in COVID-19 patients are positively associated with both circulating N antigen levels and COVID-19 severity [88,91]. Additionally, tissue damage biomarkers such as serum amyloid A (SAA) and ferritin are significantly elevated in COVID-19 patients with detectable N antigen, whereas procalcitonin (PCT) levels remain comparable to those of antigenemia-negative individuals [89]. The human humoral fluid-phase pattern recognition molecule (PRM) long pentraxin 3 (PTX3), a key anti-inflammatory component in innate immunity, has been shown to bind to the NTD of N proteins [92]. Although PTX3 does not effectively interfere with the viral life cycle, its serum levels show significant correlations with clinical inflammatory markers such as IL-6, IL-8, IL-10, CRP, total leukocyte count, neutrophil-to-lymphocyte ratio, and ferritin [92,93]. These associations support the idea that PTX3 binding to the N protein may contribute to disease severity in COVID-19 patients.

In addition to the lungs, the N protein has also been detected in various other organs, including the kidneys, heart, liver, colon, and small intestine, where it potentially contributes to both local and systemic inflammation (Figure 2) [94]. Remarkably, N protein in the hippocampus has been found to induce Tau phosphorylation, leading to cognitive impairment and associations with Alzheimer’s disease pathology [95]. This is attributed to the recruitment of Tau into SGs by N protein. However, the expression of SUMO2 can positively ameliorate N protein-induced Tau phosphorylation by directly promoting the SUMOylation of Tau or indirectly regulating the target kinase activity [95].

Although NSPs and accessory proteins are involved in modulating host inflammation, their primary role is immune evasion rather than the activation of inflammatory responses or direct association with sysmetic inflammation [4,96]. In contrast to the membrane-bound spike protein and the envelope protein embedded in the viral particle, the N protein is cytoplasmic and undergoes minimal glycosylation, making it more accessible to intracellular space and facilitating broad innate immune activation that contributes to systemic inflammation [45]. Therefore, the N protein plays a distinct pro-inflammatory role in host immune responses.

### 3.2. N Protein and Adaptive Immunity

Antibody responses to N protein have been shown to correlate with various demographic and clinical factors, such as anti-S antibody levels, smoking status, education level, income level, race/ethnicity, and age [97]. B-cell responses targeting the N protein can persist for up to six months following infection with COVID-19 [98]. Immunoglobulin M (IgM) specific to N protein peaks at around day nine after symptom onset, followed by a class switch to immunoglobulin G (IgG) [99]. Serum levels of anti-N antibodies remain detectable in convalescent individuals for up to two months post infection, although they decline significantly in most individuals after three months [100]. Notably, the serum level of IgG specific to N protein in recovered patients is positively associated with age [101]. Additionally, SARS-CoV-2 infection elicits durable cellular responses. Polyfunctional memory T cells remain detectable up to 12 months post infection [102]. During the onset of infection, the frequencies of circulating N-specific CD8^+^ and CD4^+^ T cells inversely correlate with upper airway viral loads and systemic inflammatory markers, suggesting that these N-specific T cell responses may be attributable to viral clearance prior to seroconversion [103]. CD8^+^ T cell and CD4^+^ T cell responses following natural infection have been reported to be stable for over 8 months [104]. CD8^+^ T cells targeting N protein are not only immunodominant but also more persistent than CD8^+^ T cells targeting S, E, and the matrix [105]. Notably, N-specific T cells have demonstrated protective roles in SARS-CoV-2 infection [106]. Recovered COVID-19 individuals carrying the human leukocyte antigen (HLA)-B7^+^ exhibit dominant CD8^+^ T cell responses targeting an epitope located in N-NTD [107]. High naive precursor frequency and promiscuous T cell receptor (TCR) αβ pairing in B7/N_105_^+^ CD8^+^ T cells support CD8^+^ T cell responses to immunodominant SARS-CoV-2 N epitopes. Moreover, CD8^+^ T cells recognizing N epitopes can be cross-reactive with circulating HCoV-OC43 and HCoV-HKU1 [107,108].

### 3.3. N Protein and Long COVID

**Figure 3:**
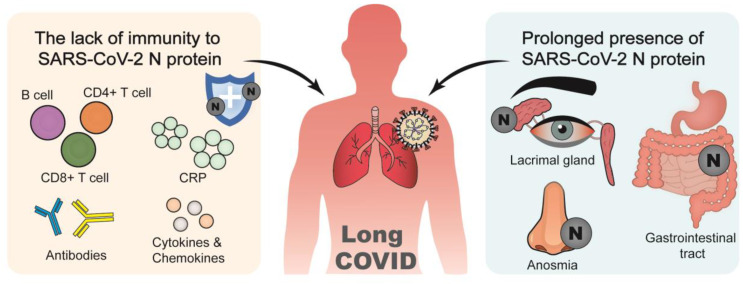
The interaction between long COVID and SARS-CoV-2 N protein.

Long COVID, or post-acute sequelae of SARS-CoV-2 infection (PASC), refers to a broad spectrum of new symptoms continuing for weeks or months after SARS-CoV-2 infection. The sequelae include cardiac arrhythmias, chronic respiratory failure, neuropsychiatric symptoms, and gastrointestinal symptoms [109,110]. Long COVID may be linked with ineffective clearance and prolonged immune activation by the adaptive immune system. Absence of increased immunity against the N protein, combined with its potent pro-inflammatory effect to stimulate cytokine and chemokine pathways, presumably contributes to the pathogenesis of long COVID (Figure 3) [111].

The N protein acts as a PAMP that activates multiple innate inflammatory signaling pathways (Figure 1), playing a significant role in triggering the cytokine storm, which is increasingly implicated in the development of long COVID. In patients with long COVID, a significantly increased number of N protein-specific CD4^+^ and CD8^+^ T cells that produce TNF-α and IL-2 has been observed [112]. It has been reported that cytokine storms characterized by elevated plasma IL-1β, IL-6, and TNF cytokine levels are associated with acute sequelae of COVID-19 [113]. Clinical inflammatory markers linked with N protein levels, such as elevated CRP and sustained levels of cytokines like IL-6, have also been associated with prolonged recovery time in COVID-19 patients [114]. Notably, findings from a systematic review and meta-analysis indicate the increased IL-6 levels alone are correlated with long COVID-19 [115]. Additionally, lower levels of initial anti-N IgG are associated with longer COVID symptom duration [110]. Antibodies specific to N protein and anti-N IgA concentrations have been detected in PASC patients who display higher humoral response during the acute stage, along with elevated levels of CD8^+^ T cells [116,117]. Moreover, individuals with PASC tend to experience a faster reduction in the prevalence of N-specific IFN-producing CD8^+^ T cells [104]. Specific immunological properties, marked by heightened CD4^+^ T cells and declined CD8^+^ memory T cells activation against the N-CTD, have been observed in neurologic PASC patients [118,119]. Therefore, N protein plays an active immunopathological role, spanning from acute infection to chronic post-acute sequelae.

The prolonged existence of N protein was detected in patients who succumbed to viral pneumonia, even 50 days after infection onset, indicating that the N protein does not degrade quickly but persists long after infection (Figure 3) [120]. Apart from the respiratory and immune system, N protein also impacts other tissues and disrupts hormonal balance at the post-infectious stage. N protein was identified in the conjunctiva, trabecular meshwork, and iris cells of a COVID-19 patient who was later diagnosed with acute angle-closure glaucoma and cataract after recovery [121]. The pathogenic mechanism of dacryoadenitis is potentially linked to SARS-CoV-2 as well, as inflammatory cells surrounding the lacrimal gland in a COVID-19 patient showed immunoreactivity to the N protein [122]. Anosmia, a long-lasting COVID-19 symptom accompanied by signs of local inflammation in the human olfactory epithelium, is putatively attributed to the presence of the N protein [123,124]. The possible mechanism is that interference with LLPS by the N protein, which suppresses the innate immune responses, contributes to long COVID [125]. The N protein has also been found in the gastrointestinal tract, and its presence is associated with morphological alterations of microvilli in the small intestinal wall, leading to bleeding ulceration in a patient who had recovered from COVID-19 [126]. Failure to clear N proteins may result in persistent immune responses with ongoing immune dysregulation, promoting the progression from acute infection to chronic sequelae.

### 3.4. N Protein and Pre-Existing Inflammatory Diseases

As SARS-CoV-2 induces severe inflammation in the host after infection, patients with pre-existing inflammatory disorders are at a higher risk of increased rates of hospitalizations and mortality [127,128]. Compared with healthy individuals, patients diagnosed with diabetes (type 1 and 2) or obesity do not have increased susceptibility to SARS-CoV-2 infection but do show elevated hospitalization rates and greater disease severity [129]. N protein has been identified in various organs (Figure 2), where it has the potential to exacerbate or interfere with existing inflammatory diseases [94]. The serum anti-N IgG titer of pneumonia patients was higher than that of the asymptomatic group after 6 months [101]. Therefore, the immunodominant function of the N protein may drive prolonged inflammation and contribute to poor outcomes in patients with inflammatory conditions such as diabetes. Patients with type 2 diabetes (T2D) display abnormally high levels of metabolites such as trimethylamine N-oxide (TMAO), which are associated with gut dysbiosis and can contribute to chronic inflammatory and degenerative diseases [130,131]. Interestingly, N proteins show synergistic effects with TMAO on NLRP3 inflammasome activation. This activation is regulated by the sterol regulatory element-binding proteins (SREBP) cleavage–activating protein (SCAP) pathway and associated with intracellular cholesterol homeostasis [132]. Hyperphosphatemia, a common complication of chronic kidney disease, drives the gathering of inorganic phosphate in the blood, which amplifies N protein-induced inflammation through the SCAP-SREBP pathway, facilitating the activation of the NLRP3 inflammasome [133]. The N protein also undergoes LLPS, during which an RNA-binding protein called endogenous transactive response-binding protein 43 kDa (TDP-43) is incorporated into the biomolecular condensate [134]. Since the presence of pathological glial cytoplasmic inclusions precedes fatal neurodegenerative disorders, this finding suggests a potential pathological association between chronic neurodegeneration and N proteins [134,135]. Notably, the N protein exhibits critical function in impeding tumor proliferation and metastasis in colon and kidney cancers, an effect mediated through both its NTD and CTD [136]. It also destabilizes the expression of pyruvate kinase M (PKM), an enzyme that promotes tumor proliferation and metastasis, by interacting with another key regulator, Y-box binding protein 1 (YBX1), through the CTD domain, thereby facilitating the G3BP1-mediated recruitment of PKM mRNA into SGs [136]. Additionally, the N protein exacerbates pre-existing cognitive impairment in mice, suggesting that individuals with underlying neurodegenerative diseases may experience a worsening of their condition following exposure to SARS-CoV-2 and the development of COVID-19 [95].

## 4. Conclusions and Future Work

In summary, the N protein is essential to the SARS-CoV-2 viral life cycle, including viral replication, translation, and assembly, and contributes to viral pathogenesis by interacting with various immune regulatory pathways. Compared to other SARS-CoV-2 viral proteins, the N protein functions as an immunodominant driver due to its abundant and persistent presence in host cells, along with its extensive interactions with multiple immune regulatory pathways. As a result, it plays a central role in both acute immune activation and the development of chronic post-acute sequelae.

Further elucidation of the comprehensive mechanisms of N protein in viral replication and SARS-CoV-2 pathogenesis is requisite for reducing virulence and controlling acute disease progression. Remarkably, N protein also interacts with various pre-existing inflammatory diseases, including diabetes, obesity, and cancer. Due to its prolonged presence and immunogenicity in infected hosts, the N protein is also implicated in long COVID. Unraveling the mechanisms underlying the persistent effects of PASC will provide a crucial foundation for the development of advanced antiviral therapies. There is also an urgent need to understand the long-term immune responses to N protein and to identify strategies for eliminating its persistent presence, not only in the respiratory system but also in other susceptible tissues.

Although mRNA-based COVID-19 vaccines targeting the S gene have been successfully deployed worldwide and proven to be effective, the evolution of SARS-CoV-2 due to immune escape mutations makes it challenging to maintain adequate herd immunity. Given that N protein interacts with various host factors to drive viral pathogenesis and exhibits higher sequence conservation than the S protein, studies using various vaccine platforms have shown the usefulness of including N protein in vaccine composition to broaden adaptive immune responses [137,138]. A proof-of-concept study has also shown that an anti-N monoclonal antibody (mAb) can block complement hyperactivation induced by the N protein [139]. Another study demonstrated that anti-N mAb reduced viral load in the lungs of infected mice when given as a prophylactic treatment [140]. Thus, further work could also focus on developing new therapeutic treatments for COVID-19 by using mAbs, nanobodies, or small molecules to target the N protein.

## Data Availability

Not applicable.

## Abbreviations

The following abbreviations are used in this manuscript:

AP3B1	Adaptor-related protein complex 3 subunit beta 1
ASC	Apoptosis-associated speck-like protein containing CARD
ASCC3	Activating signal co-integrator 1 complex subunit 3
CFTR	Cystic fibrosis transmembrane conductance regulator
COVID-19	Coronavirus disease 2019
CoVs	Coronaviruses
CRP	C-reactive protein
CTD	C-terminal domain
dsRNA	Double-stranded RNA
E	Envelope protein
ERGIC	Endoplasmic reticulum–Golgi intermediate compartment
ERK1/2	Extracellular signal-regulated kinases 1 and 2
G3BP1	GTPase-activating protein-binding protein 1
GADD34	Growth arrest and DNA damage-inducible 34
GSDMD	Gasdermin D
GSK-3	Glycogen synthase kinase-
HCoV	Human coronavirus
HDAC6	Histone deacetylases 6
HERC5	HECT and RLD domain containing E3 ubiquitin protein ligase 5
HLA	Human leukocyte antigen
ICU	Intensive care unit
IDRs	Intrinsically disordered regions
IFN-1	Type I interferon
IgG	Immunoglobulin G
IgM	Immunoglobulin M
IKKα	IκB kinase-α
IL	Interleukin
IMPDH2	Inosine monophosphate dehydrogenase 2
IP-10	IFN-gamma-inducible protein 10 kD
IRF	Interferon regulatory factor
ISG15	IFN-stimulated gene 15
JAK-STAT	Janus kinase-signal transducer and activator of transcription
KPNA	Karyopherin alpha
KPNB1	Karyopherin beta 1
LKR	Linking region
LLPS	Liquid–liquid phase separation
M	Membrane protein
MAPK	Mitogen-activated protein kinase
MASP-2	Mannan-binding lectin serine protease 2
MAVS	Mitochondrial antiviral signaling
MDA5	Melanoma differentiation-associated gene 5
MERS-CoV	Middle East respiratory syndrome coronavirus
MHV	Mouse Hepatitis Virus
MIS-C	Multisystem inflammatory syndrome
N	Nucleocapsid
NF-κB	Nuclear factor kappa B
NFKB1	NF-κB subunit 1
NFKBIA	NF-κB inhibitor alpha
NLRP3	NLR family pyrin domain containing-3
NLRs	NOD like receptors
NMD	Nonsense-mediated mRNA decay
NOD	Nucleotide-binding oligomerization domain proteins
NSPs	Nonstructural proteins
NTD	N-terminal domain
ORFs	Open reading frames
PAMPs	Pathogen-associated molecular patterns
PASC	Post-acute sequelae of SARS-CoV-2 infection
PCT	Procalcitonin
PDE4	Phosphodiesterase 4
PKM	Pyruvate kinase M
PLpro	Papain-like protease
poly(I:C)	Polyinosinic: polycytidylic acid
PRRs	Pattern recognition receptors
PTX3	Pentraxin 3
RAGE	Glycation end products
RAN	Ras-related nuclear protein
RdRp	RNA polymerase
RIG-I	Retinoic acid-inducible gene I
RISC	RNA-induced silencing complex
RLRs	RIG-I-like receptors
RNAi	RNA interference
RNP	Ribonucleoprotein
S	Spike protein
SAA	Serum amyloid A
SARS-CoV-2	Severe acute respiratory syndrome coronavirus 2
SARSr-CoV	SARS-related coronavirus
SCAP	SREBPs cleavage-activating protein
SGK1	Serum glucocorticoid regulated kinase 1
SGs	Stress granules
siRNA	Small interfering RNAs
SL	Stem-loop
SNX8	Sorting nexin 8
SR	Ser/Arg
SREBPs	Sterol regulatory element-binding proteins
SUMO	Small ubiquitin-like modifiers
T1D	Type 1 diabetes
T2D	Type 2 diabetes
TBK1	Tank-binding kinase 1
TCR	T cell receptor
TDP-43	Transactive response-binding protein 43 kDa
Th1	T helper type 1
TLR2	Toll-like receptor 2
TMAO	Trimethylamine N-oxide
TNF	Tumor necrosis factor
TNFR2	Tumor necrosis factor receptor 2
TRIM25	Tripartite motif protein 25
TRIM6	Tripartite motif protein 6
UBC9	Ubiquitin-conjugating enzyme 9
UPF1	UP-frameshift-1
VOCs	Variants of concern
YBX1	Y-box binding protein 1
